# Chlorophyll derivatives enhance invertebrate red-light and ultraviolet phototaxis

**DOI:** 10.1038/s41598-017-03247-1

**Published:** 2017-06-13

**Authors:** Andrea Degl’Innocenti, Leonardo Rossi, Alessandra Salvetti, Attilio Marino, Gabriella Meloni, Barbara Mazzolai, Gianni Ciofani

**Affiliations:** 1 Center for Micro-BioRobotics, Istituto Italiano di Tecnologia, Viale Rinaldo Piaggio 34, 56025 Pontedera (Pisa), Italy; 2 Department of Clinical and Experimental Medicine, Università di Pisa, Via Alessandro Volta 4, 56126 Pisa, Italy; 3 Smart Bio-Interfaces, Istituto Italiano di Tecnologia, Viale Rinaldo Piaggio 34, 56025 Pontedera (Pisa), Italy; 4 The BioRobotics Institute, Scuola Superiore Sant’Anna, Viale Rinaldo Piaggio 34, 56025 Pontedera (Pisa), Italy; 5 Department of Mechanical and Aerospace Engineering, Politecnico di Torino, Corso Duca degli Abruzzi 24, 10129 Torino, Italy

## Abstract

Chlorophyll derivatives are known to enhance vision in vertebrates. They are thought to bind visual pigments (*i.e*., opsins apoproteins bound to retinal chromophores) directly within the retina. Consistent with previous findings in vertebrates, here we show that chlorin e_6_ — a chlorophyll derivative — enhances photophobicity in a flatworm (*Dugesia japonica*), specifically when exposed to UV radiation (λ = 405 nm) or red light (λ = 660 nm). This is the first report of chlorophyll derivatives acting as modulators of invertebrate phototaxis, and in general the first account demonstrating that they can artificially alter animal response to light at a behavioral level. Our findings show that the interaction between chlorophyll derivatives and opsins virtually concerns the vast majority of bilaterian animals, and also occurs in visual systems based on rhabdomeric (rather than ciliary) opsins.

## Introduction

In order to sense light, animals rely on specialized macromolecules, namely photoreceptor proteins; these are required for the initial sensory transduction event that eventually leads to vision. At least two groups of photoreceptor proteins are known so far, and a third one has recently been proposed^1^. The most diversified and widely distributed type of photoreceptor protein is opsins. Opsins belong to a multifamily of membrane proteins known as G protein-coupled receptors (GPCRs), or seven-transmembrane receptors. GPCR genes are extremely abundant — among others — in animal and fungal genomes, and GPCRs constitute the most common target for drugs, *cf*., ref. 2.

Opsins bind retinal, a chromophore that captures light and transfers energy to the apoprotein; together they form a visual pigment. Photons within a certain stretch of wavelengths are trapped: absorption range ultimately depends on the structure of retinal, as well as on the way this contacts its binding pocket within the opsin (reviewed in ref. 3). However, there are a few instances where the spectral tuning of an opsin does not depend solely on these factors.

In fact, the meso-bathypelagic fish *Malacosteus niger* is capable of seeing far-red light in spite of the absorption spectrum of its opsins, which are green-tuned and display negligible absorption already at λ = 650 nm. Through its diet, the fish concentrates chlorophyll derivatives (CDs) within its retina, in the same anatomic compartment that contains visual pigments, the rod outer segment (ROS). Specifically, *M. niger* accumulates demetallated and defarnesylated derivatives of bacteriochlorophylls C and D^4–8^.

**Figure 1:**
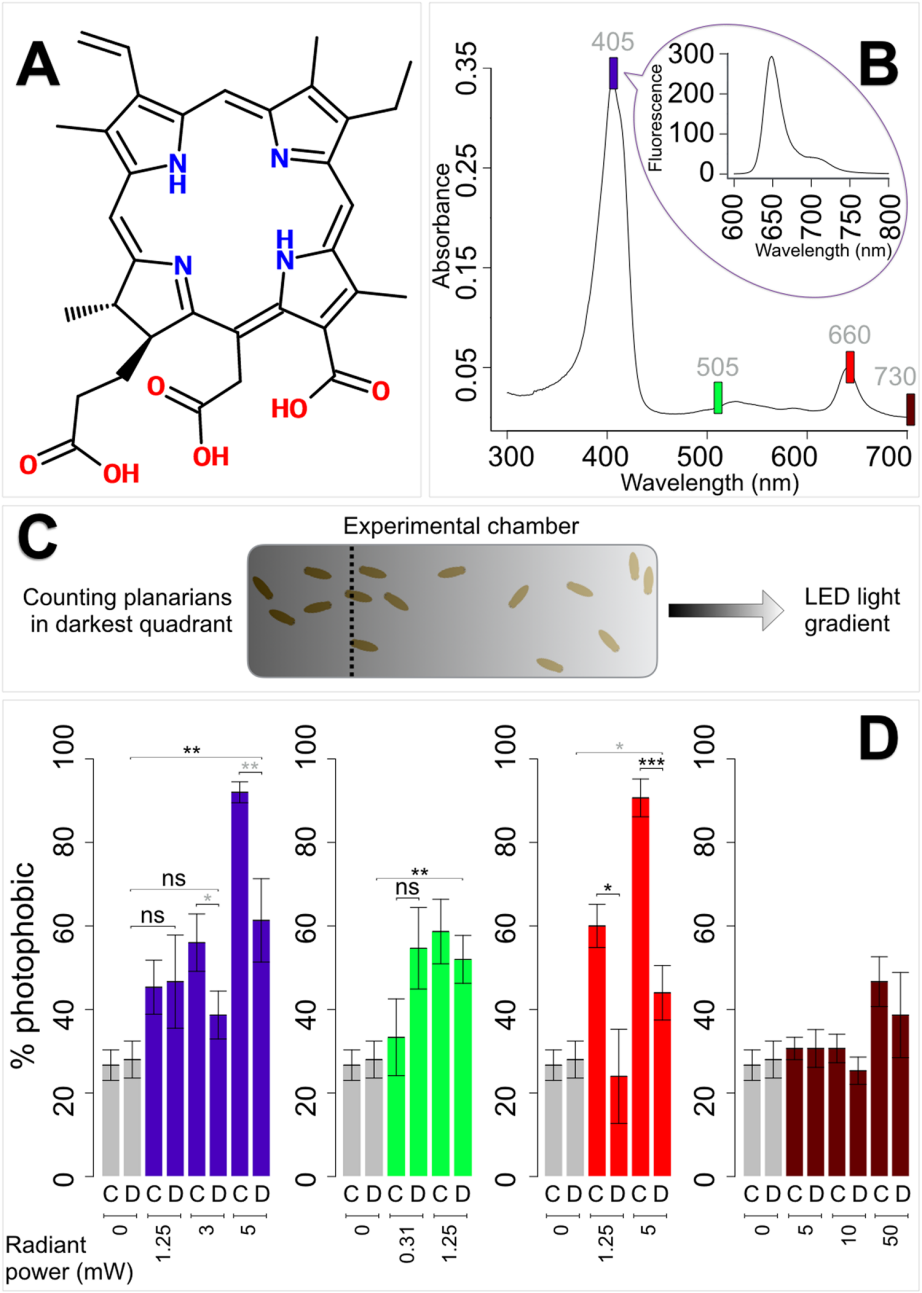
Chlorin e_6_ enhances UV and red-light avoidance in *Dugesia japonica*. (**A**) Skeletal formula of chlorin e_6_. (**B**) Absorption and emission (smaller graph, excitation wavelength ~405 nm) spectra of chlorin e_6_ dissolved in 1% v/v dimethyl sulfoxide; colored boxes approximate wavelength positions (exact values in gray, nm) for the tested colors (left to right: UV, cyan, red, NIR). (**C**) Representation of the experimental chamber used for behavioral experiments, with LED light diffusing from the right edge; animals found within the darkest quadrant (area delimited by the vertical dashed line) after two minutes of light exposure were counted as photophobic. (**D**) Bar charts reporting the percentage of photophobic animals, either treated with chlorin e_6_ (**C**) or plain dimethyl sulfoxide (**D**), after exposure to light of different wavelengths (charts from left to right: UV, cyan, red, NIR) at different radiant power. Grey bars illustrate control experiments with no light stimulus provided. Grey asterisks indicate significance for unpaired one-tailed T-test; black asterisks indicate significance for unpaired one-tailed T-test and two-tailed Mann Whitney U-test. The number of asterisks specifies significance for different p-value threshold of the unpaired one-tailed T-test (*for p < 0.05, **for p < 0.01, ***for p < 0.001); such threshold, for the two-tailed Mann Whitney U-test, is invariably p < 0.05. n = 5, 15 specimens *per* experiment, 75 animals *per* class; error bars report standard error of the mean.

Following this natural example, some studies explored the possibility of CDs being modulators of vertebrate vision. A handful of CDs were tested as candidate photosensitizers of purified bovine rhodopsin (an opsin): one of them, a substituted chlorin named chlorin e_6_ (Ce_6_, Fig. 1), proved to be a potent bleaching agent for the protein upon exposure to red light^9^. Together with metal ions, it could also stabilize rhodopsin against thermal denaturation^2^. Another study showed that living rod cells, extracted from the salamander *Ambystoma tigrinum*, respond better to red light if exposed to Ce_6_^10^. When injected intravenously in a mouse model Ce_6_ could rapidly accumulate in the ROS, and caused improved electroretinogram responses to red (>640 nm) and blue (456 ± 30 nm) wavelengths^11^. Intravenous administration of Ce_6_ (or related compounds) in macaque and rabbit also led to bioaccumulation in the retina^12–14^. Moreover, photosensitization is reported to occur in human subjects as a side effect of photodynamic therapy, a medical treatment in which photosensitizers are used in combination with localized light to achieve selective cell death, *cf*., ref. 9. There is also anecdotal evidence for human night vision improvement following Ce_6_ exposure. CDs are likely to bind rhodopsins directly^2, 4, 6, 9, 10^, within a binding pocket that differs from the one that accommodates retinal^15, 16^.

The study of the role of CDs in vision is of profound interest both for basic and applied biology: owing to the diffusion of GPCRs, as well as their importance as drug targets, the topic deserves attention from the biomedical community, even beyond the field of ophthalmology. However, in spite of its potential relevancy, the stream of research pivoting on the role of CDs as allosteric modulators of opsins remained predominantly dormant in the last years; indeed, a number of crucial points remain to be investigated: in particular, here we aim at addressing whether the interaction between opsins and CDs can be considered a general phenomenon throughout the animal kingdom, and to show at a behavioral level that CDs can affect sight. Finally, with just a few CDs being investigated so far, we propose *Dugesia japonica* (an easy-keeper flatworm with a distinct photophobic behavior and remarkable regeneration capabilities^17–19^) as an effective mean to test the effects of different CDs on vision.

## Results

Foremost, we confirmed emission and absorption spectra for Ce_6_ dissolved in 1% dimethyl sulfoxide (DMSO).

For absorption, we found a first 405 nm peak in the UV range, a minor peak in the green range (λ = 528 nm), and a second distinct peak in the red at λ = 640 nm. A single emission peak was observed around 649 nm of wavelength, in the red. Absorption and emission spectra (excitation λ ~ 405 ± 5 nm) are reported in Fig. 1.

In order to assess whether CDs — specifically Ce_6_ — could diffusely affect animal vision, we chose the planarian *D. japonica* as a non-vertebrate animal model. To test for possible Ce_6_-mediated photophobic (or photophilic) behaviors, we deployed a behavioral assay consisting of a water-filled chamber in which planarians could move freely; on one side of such container, an array of LEDs of different wavelength (UV, cyan, red and NIR) was positioned. Radiant power (Φ_e_) was adjustable. Measured flux densities for LEDs at Φ_e_ = 5 mW were 72.2 mW/cm^2^ (UV), 143.5 mW/cm^2^ (cyan), 90.0 mW/cm^2^ (red) and 109.2 mW/cm^2^ (NIR).

Prior to behavioral tests, groups of 15 specimens were soaked in either Ce_6_ in 1% DMSO or 1% DMSO only (for control experimental classes). Immediately after treatment, animals were rinsed and briefly exposed to the light of a single LED at fixed Φ_e_. Animals residing in the darker side of the chamber were counted as photophobic.

For significance threshold set at 0.05, Ce_6_-treated animals show enhanced photophobicity for UV and red light with respect to controls: for UV, a gain of function (*i.e*., increased light avoidance) is observed at 3 and 5 mW of Φ_e_ (p-values for unpaired one-tailed T-test, respectively, 0.04 and 0.009); for red, increased photophobicity occurs at 1.25 and 5 mW of Φ_e_ (with p-values for unpaired one-tailed T-test of 0.01 and 0.0002, and p-values for two-tailed Mann Whitney U-test of 0.04 and 0.01, respectively). No significant alterations of light response were detected for cyan and NIR. Results are summarized in Fig. 1.

To prove a direct involvement of planarian visual system in the observed light-avoidance behavior, we repeated experiments for red light at 5 mW of Φ_e_ using visually impaired animals, *i.e*., either head-regenerating (decapitated) worms or opsin-less animals with severely reduced eyes (obtained *via* RNA interference, RNAi).

**Figure 2:**
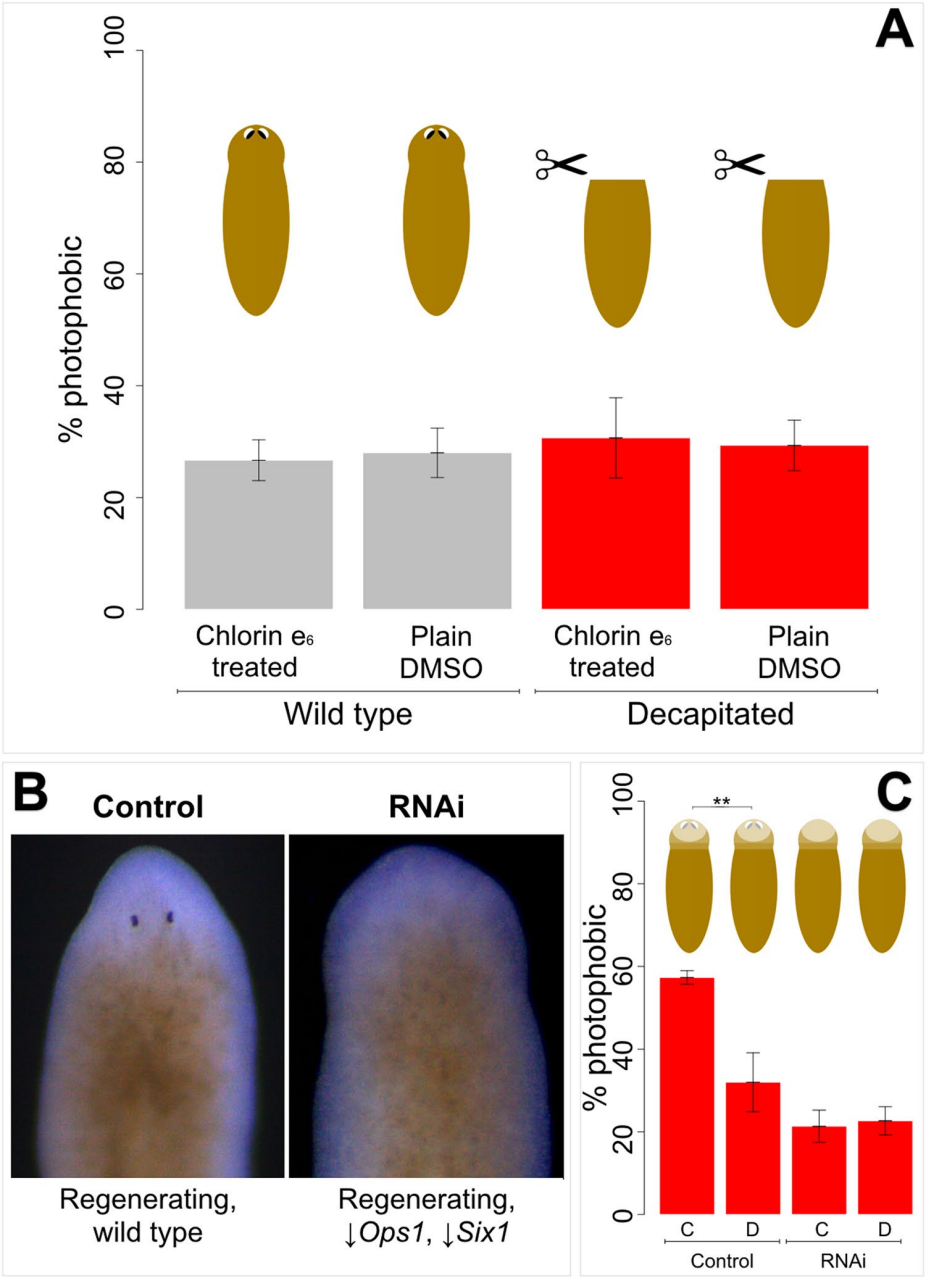
Vision is required for chlorin e_6_-triggered red-light avoidance. (**A**) bar charts reporting the percentage of photophobic animals, either treated with chlorin e_6_ or plain dimethyl sulfoxide, in absence of light stimulus (Wild type, grey bars) or after exposure to red light at 5 mW of radiant power (Decapitated, red bars). n = 5, 15 specimens *per* experiment, 75 animals *per* class; error bars report standard error of the mean. (**B**) Representative pictures of regenerating animals following RNA interference (RNAi, reported as ↓*Ops1*, s*Six1*) or simple incubation on ice (Control). RNAi individuals had eyeless phenotype, and a 99% opsin reduction (assessed *via* qPCR). (**C**) Bar charts reporting the percentage of photophobic animals (RNAi or Control), either treated with chlorin e6 (**C**) or plain dimethyl sulfoxide (**D**), after exposure to red light at 5 mW of radiant power. Two asterisks indicate significance for unpaired one-tailed T-test (for p < 0.01); the difference shown is also significant for two-tailed Mann Whitney U-test (for p < 0.05). n = 5, 15 specimens *per* experiment, 75 animals *per* class; error bars report standard error of the mean.

Decapitated Ce_6_-treated animals show no relevant behavioral differences with respect to decapitated control groups; their behavior is also indistinguishable from that of wild-type no-light control specimens (either Ce_6_-treated or plain DMSO-treated, Fig. 2). Similarly, RNAi individuals display no gain of function when treated with Ce_6_, compared with untreated RNAi animals. However, a significantly (α = 0.05) increased photophobicity is observed when non-RNAi planarians (a negative control for RNAi procedures) are treated with Ce_6_ (p-value for unpaired one-tailed T-test of 0.004, and p-value for two-tailed Mann Whitney U-test of 0.02), relative to non-RNAi non-Ce_6_-treated controls (Fig. 2).

In addition to a general evaluation of the morphology for RNAi animals, the reduction of opsin transcript was evaluated *via* qPCR.

RNAi specimens displayed severely reduced or absent eyes, and opsin mRNA concentrations equal to 1% of wild-type values (Fig. 2).

We finally repeated behavioral experiments for red at Φ_e_ = 5 mW in wild-type planarians belonging to an asexual population of *D. gonocephala* s.l.

Similarly to *D. japonica*, *D. gonocephala* s.l. specimens display increased light avoidance following Ce_6_ exposure (relative to controls, Supplementary Fig. S1). For α = 0.05, the p-value for an unpaired one-tailed T-test amounts to 0.004, and the one for a two-tailed Mann Whitney U-test equals 0.04.

## Discussion

Our spectroscopic analyses were substantially aimed at confirming that a water solution containing 1% DMSO is an acceptable solvent for Ce_6_. Distinct absorption and emission peaks were obtained, at wavelengths that are in line with previous findings^20^.

With this work, we proved that flatworms acquire increased UV and red-specific photophobicity upon Ce_6_ treatment. As visually impaired specimens do not display any obvious behavioral alteration after exposure to Ce_6_, strictly speaking we conclude that such enhancement represents a *bona fide* photosensitization. Given the existing body of literature supporting an unambiguous role for CDs in vision, we believe it is conservative to consider the observed effects to be directly mediated by sight.

Planarians were used because they constitute a convenient representative of invertebrate bilaterians. The planarian eyespot possesses all key features of the metazoan visual system, featuring opsins, retinal, conserved pathways for development and sensory transduction, as well as specialized cell types like photoreceptor neurons and pigmented cells^21–28^. Still, it displays remarkable differences with respect to the vertebrate eye: importantly, photoreceptor cells are rhabdomeric rather than ciliary, and contain r-opsins instead of c-opsins^27, 28^; since the two opsin types differ in their downstream signaling cascades (G protein included), and provided that Ce_6_ is thought to bind bovine rhodopsin within its G protein-binding pocket^15, 16^, our results broaden the relevance of this molecular interaction. In fact, the UV and red-specific visual enhancement we detected in planarians firmly recapitulates the observations in mice from Washington *et al*.^11^; as they noticed, wavelengths causing visual gain of function correspond to peaks in the absorption spectrum of Ce_6_. Furthermore, we showed that soaking animals in a Ce_6_-containing solution for a few minutes (see section “Photophobicity assay” in “Methods”) was sufficient to elicit drastic changes in visual perception. Similarly, several groups found that in vertebrate models Ce_6_ promptly reaches (within minutes) the ROS, even if the substance is administered systemically^11–14^. All in all, Ce_6_ seems to have rapid uptake and to cause opsin spectral shift at specific wavelengths in invertebrates as much as in vertebrates.

With roughly 66,000 extant species described, vertebrates (subphylum Vertebrata) are widely regarded as a successful clade of animals^29^. Yet, compared to the entire animal kingdom they represent a minority: in fact, not less than 95% of known living animals are invertebrate^29^.

Formally, up to now any evidence for CDs as modulators of opsin function was confined to jawed vertebrates (superclass Gnathostomata, ~ 60,000 extant species known^29^). By providing the first example of a protostomian invertebrate whose vision could be enhanced by CDs, we extended such limit to the majority of bilaterians, Nephrozoa. The molecular basis of this interaction is therefore likely to be ancient, possibly even predating Cambrian explosion, the age in which proper (image-forming) eye and sight are thought to have appeared (reviewed in refs 30 and 31). These mechanisms are also expected to be general, as it is unlikely — we believe — that we basically found by chance a genre (*Dugesia* spp.) that exhibits as a peculiarity improved visual performances when treated with Ce_6_. Instead, our results support the existence of a common mechanism that readily triggers a spectral shift in opsins whenever the photoreceptor neuron is exposed to CDs. Such a dynamic would not necessarily be conserved as a consequence of selective pressure on the capability to react to CDs, rather because CDs interfere with a pre-existing interaction that serves a crucial function of the photoreceptor neuron. For instance, this view is compatible with CDs being allosteric modulators of opsins, which contact the protein within a conserved pocket that already acts as a binding site for the G protein.

Our work provides the first structured evidence of CDs modulating response to light at a behavioral level. The number of individuals we were able to include in this study is in the order of thousands: hence, our method also constitutes a mean to rapidly but robustly screen the several untested CDs as enhancers of vision.

## Methods

### Animals

The main model species of the study was the planarian worm *Dugesia japonica* (Platyhelminthes, Dugesiidae); a second *taxon* we used was an asexual population of planarians coming from the Zambra creek, Calci (Pisa), central Italy; this can be attributed to *D. gonocephala* group on the basis of general morphology.

Animals were reared in planarian water (distilled water with CaCl_2_ 2.5 mM, MgSO_4_ 0.4 mM, NaHCO_3_ 0.8 mM, KCl 0.077 mM) at 18 °C in dim light conditions, and fed with chicken liver (purchased from local food stores) once a week. Non-regenerating specimens within 5–8 mm of length were used for all experimental procedures, after being starved for about two weeks.

Authors declare that all animal (non-cephalopod invertebrates) experiments were performed in compliance with Italian and European law, as well as with guidelines and recommendations of the Federation of European Laboratory Animal Science Associations (FELASA).

### Chlorin e_6_ preparation

Ce_6_ powder (Santa Cruz Biotechnology sc-263067A) was dissolved in DMSO (Sigma-Aldrich D2650) to a final concentration of 10 μg/μl; the solution was left on a horizontal shaker at 250 RPM for 4 h at room temperature, then aliquoted and stored at −20 °C. At least half an hour prior to behavioral tests (see section “Photophobicity assay”), single aliquots (as needed) were diluted 1:4 in DMSO and kept at room temperature. A further 1:100 dilution in planarian water was prepared immediately prior to each behavioral assay; we used this mixture as a soaking medium for planarians (or a 1% v/v DMSO solution in planarian water for control animals), see section “Photophobicity assay”. All handling of Ce_6_ was performed in the dark.

### Spectroscopy

We evaluated absorption and emission properties of Ce_6_ when dissolved in 1% v/v DMSO. For this, we dissolved Ce_6_ powder in DMSO to a final concentration of 10 μg/μl. This mixture was left to mix on a horizontal shaker at 250 RPM for 4 h at room temperature, then diluted in DMSO-containing water to a final concentration of 3.3 ng/μl, 1% DMSO. Ce_6_ was invariably handled in a dark environment.

Spectroscopic analyses were conducted in disposable cuvettes (Fisherbrand FB55143), using an UV/VIS Lambda 25 spectrophotometer (PerkinElmer) to obtain the absorption spectrum, and a Cary Eclipse fluorescence spectrophotometer (Agilent Technologies) for generating the fluorescence spectrum.

### Photophobicity assay

A dedicated setup was built for behavioral studies (Fig. 1). A plastic experimental chamber (an empty microscope slide box, 7.2 cm × 3 cm) was externally covered with black tape; three straight lines were externally drawn to mark four equivalent quadrants (numbered from 1 to 4) along the main axis of the container. Four LEDs of different colors were mounted on a wooden stage: UV (Nichia NCSU276AT-405, λ_peak_ = 405 nm, Δλ = 12 nm), cyan (OSRAM LVCK7PJYKZ25, λ_peak_ = 505 nm, Δλ = 30 nm), red (ProLight Opto PK2N-3LME, λ_peak_ = 660 nm, Δλ = 30 nm) and NIR (OSRAM GF C8PM1.24-3S4S, λ_peak_ = 730 nm, Δλ = 30 nm); actual flux density at Φ_e_ = 5 mW was measured for each LED. LEDs pointed downwards, orthogonally to the bench top and at 5 cm from it. Prior to use, the experimental chamber was positioned so that a single LED (the one intended to be turned on) was located above the center of the shorter side of the container, on the external edge of quadrant 4 (hence, areas are named according to increasing luminosity).

Behavioral assays on *D. japonica*, aimed at quantifying negative phototaxis, were first performed on wild-type specimens, then on visually impaired individuals (see sections “Decapitation” and “RNA interference”). For wild-type animals, different light and treatment conditions were investigated (n = 5 for each class) as summarized in Table 1.

**Table 1:** Experimental classes for wild-type animals.

	Chlorin e_6_-treated	Plain DMSO control
No-light control	0	0
UV (405)	1.25, 3, 5	1.25, 3, 5
Cyan (505)	0.31, 1.25	0.31, 1.25
Red (660)	1.25, 5	1.25, 5
NIR (730)	5, 10, 50	5, 10, 50

Nominal radiant powers (mW) tested for each light condition (λ, nm) for chlorin e_6_-treated or control (exposed to plain dimethyl sulfoxide, DMSO) specimens. n = 5, 15 animals *per* experiment, 75 animals *per* class.

For each experiment, 15 planarians were soaked for 12–13 min in planarian water containing either Ce_6_ (final concentration Ce_6_ = 25 ng/µl, with 1% v/v DMSO) or only DMSO (1% v/v) for negative controls. Then, specimens were rinsed in planarian water, and transferred in the middle of the experimental chamber (between quadrant 2 and 3) in a single drop. Finally, in order to fill up the experimental chamber, 15 ml of planarian water were gently added, and immediately after a specific LED was turned on. After 2 min, individuals in quadrant 1 (regarded as photophobic) were counted.

All procedures except for counting photophobic animals were carried out in a dark environment. A dim yellow light was used for all preparatory work; this light was turned off during experiments. In order to minimize errors, control and experimental classes were alternated (*e.g*., for UV light at 3 mW of Φ_e_, each of five repetitions for Ce_6_-treated animals was followed by an experiment deploying individuals treated with plain DMSO). Between assays the experimental chamber was washed and rinsed thoroughly.

Besides *D. japonica*, wild-type *D. gonocephala* s.l. were used for behavioral tests (only for red at Φ_e_ = 5 mW, n = 5, either Ce_6_-treated or plain DMSO-treated as a control).

### Decapitation

A first category of visually impaired animals used for behavioral tests was obtained *via* decapitation. Specimens were put on moisturized Whatman 3MM chromatography paper (Sigma-Aldrich WHA3030917) under a stereomicroscope; their head was severed through a single coronal cut, immediately posterior to the auricular grooves. Animals were let to regenerate for 18–22 h (a time at which novel eyes are not yet formed), then used for photophobicity assays (n = 5) with red light at 5 mW of Φ_e_, after Ce_6_ or plain DMSO treatment.

### RNA interference

The second kind of visually impaired planarians deployed in photophobicity assays were obtained *via* RNAi against *DjOps* (GenBank accession: KP299262.1) and *DjSix1-2* (GenBank accession: AJ557022.1), which produces eyeless phenotype^25, 26^. dsRNA for the two genes was obtained as previously described^32^; primer sequences (containing T7 promoter adaptors) are reported in Table 2. At days 1, 5 and 10, planarians were fed with chicken liver paste containing *DjOps* and *DjSix1-2* dsRNA, according to the procedure described by Rouhana *et al*. At day 11 they were decapitated (as described in paragraph “Decapitation”). At days 12 and 16 they were microinjected with the same dsRNA mixture on ice-cooled glass slides, using a Nanoject microinjector (Drummond); at day 18 they underwent behavioral testing. Together with RNAi animals, wild-type controls were decapitated at day 11, injected with water at days 12 and 16, and used for behavioral assays as well at day 18. RNAi or control individuals were tested with red light at 5 mW of Φ_e_, either after Ce_6_ or plain DMSO exposure.

**Table 2:** Primers used for RNAi (dsDNA primers) and RT-qPCR (qPCR primers) experiments.

	Forward primer sequence	Reverse primer sequence
*DjOps* – dsDNA primers	**CGGATATAATACGACTCACTATAGGG**CAGGTGTTTTAGGAAATCTTCTCGTG	**CGGATATAATACGACTCACTATAGGG**TCATGAACTCTAACGGCTTTCACGAT
*DjSix1-2* – dsDNA primers	**CGGATATAATACGACTCACTATAGGG**CGTTTTCACCACACAACCATTACA	**CGGATATAATACGACTCACTATAGGG**GTACCATTGCTGTCATGATTACCTT
*DjOps* – qPCR primers	GCACAGAAAATGAATGCTTCTCATCC	GCATAAGGGGTCCATGACAACAAA

Primer sequences are reported 5′ to 3′. T7 promoter adaptor sequence is highlighted in bold.

The outcome of RNAi was evaluated visually for *DjSix1-2* (in terms of severe reduction or absence of the eye size^25, 26^), and *via* RT-qPCR for *DjOps*. qPCR primer sequences for *DjOps* are shown in Table 2.

### Data availability statement

All relevant data are included in the article and its supplementary information file.

## Electronic supplementary material

Supplementary figure S1
