# Supplementary material for: Chlorophyll derivatives enhance invertebrate red-light and ultraviolet phototaxis
Source: Sci Rep. 2017 Jun 13;7:3374. doi: 10.1038/s41598-017-03247-1 (PMC5469770; doi:10.1038/s41598-017-03247-1)
Supplement: Supplementary file 1 — Supplementary figure S1 [file 41598_2017_3247_MOESM1_ESM.pdf]

Chlorophyll derivatives enhance invertebrate red-light and ultraviolet phototaxis

Andrea Degl'Innocenti, Leonardo Rossi, Alessandra Salvetti, Attilio Marino, Gabriella Meloni, Barbara Mazzolai, Gianni Ciofani

Supplementary figure S1. Chlorin e_6 enhances red-light avoidance in *Dugesia gonocephala* s.l. Bar charts reporting the percentage of photophobic *D. gonocephala* s.l. specimens, either treated with chlorin e6 or plain dimethyl sulfoxide (DMSO), after exposure to red light at 5mW of radiant power. Two asterisks indicate significance for unpaired one-tailed T-test (for $p < 0.01$); the difference shown is also significant for two-tailed Mann Whitney U-test (for $p < 0.05$). $n = 5$, 15 specimens *per* experiment, 75 animals *per* class; error bars report standard error of the mean.

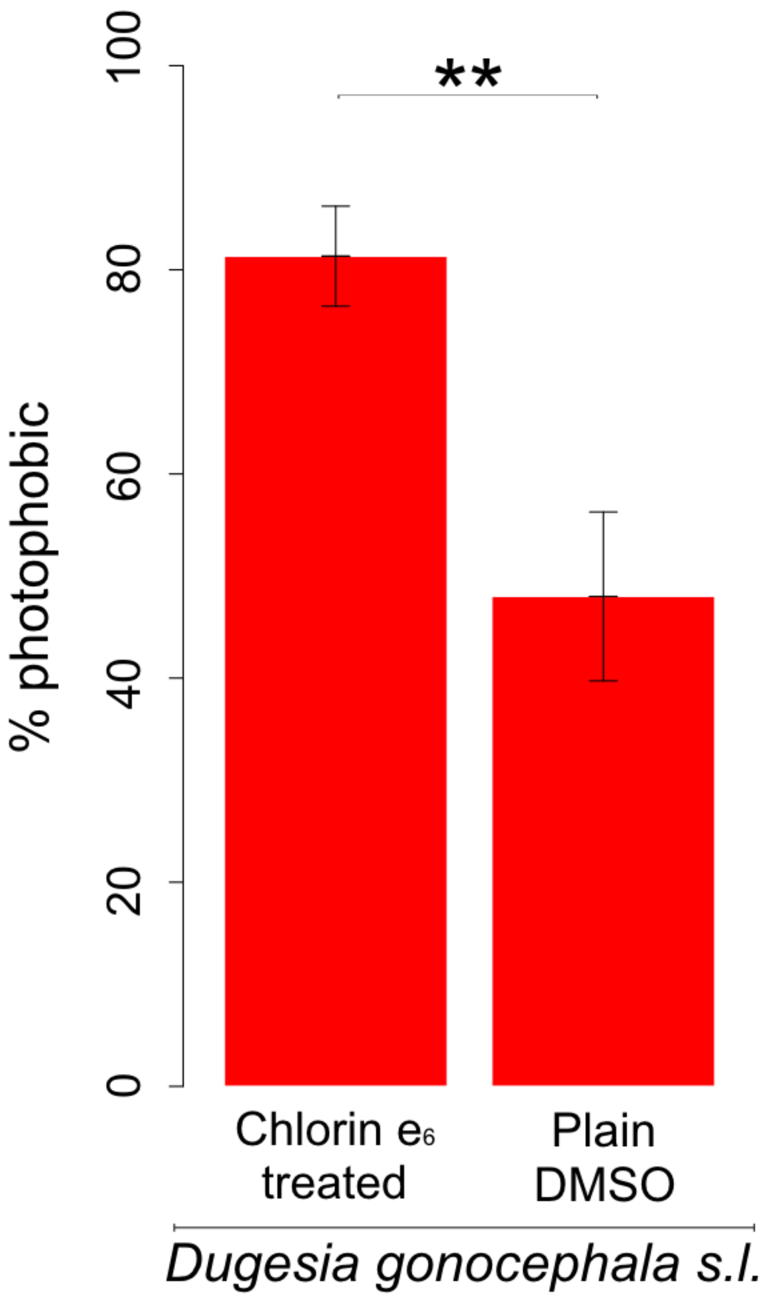

% photophobic
100
80
60
40
20
0
**
Chlorin e6 treated
Plain DMSO
Dugesia gonocephala s.l.
